# Cardiorespiratory factors related to the increase in oxygen consumption during exercise in individuals with stroke

**DOI:** 10.1371/journal.pone.0217453

**Published:** 2019-10-09

**Authors:** Kazuaki Oyake, Yasuto Baba, Nao Ito, Yuki Suda, Jun Murayama, Ayumi Mochida, Kunitsugu Kondo, Yohei Otaka, Kimito Momose

**Affiliations:** 1 Department of Physical Therapy, School of Health Sciences, Shinshu University, Matsumoto, Nagano, Japan; 2 Department of Rehabilitation Medicine, Tokyo Bay Rehabilitation Hospital, Narashino, Chiba, Japan; 3 Department of Rehabilitation Medicine I, School of Medicine, Fujita Health University, Toyoake, Aichi, Japan; Swansea University, UNITED KINGDOM

## Abstract

**Background:**

Understanding the cardiorespiratory factors related to the increase in oxygen consumption ($\dot{V}O_{2}$) during exercise is essential for improving cardiorespiratory fitness in individuals with stroke. However, cardiorespiratory factors related to the increase in $\dot{V}O_{2}$ during exercise in these individuals have not been examined using multivariate analysis. This study aimed to identify cardiorespiratory factors related to the increase in $\dot{V}O_{2}$ during a graded exercise in terms of respiratory function, cardiac function, and the ability of skeletal muscles to extract oxygen.

**Methods:**

Eighteen individuals with stroke (aged 60.1 ± 9.4 years, 67.1 ± 30.8 days poststroke) underwent a graded exercise test for the assessment of cardiorespiratory response to exercise. The increases in $\dot{V}O_{2}$ from rest to first threshold and that from rest to peak exercise were measured as a dependent variable. The increases in respiratory rate, tidal volume, minute ventilation, heart rate, stroke volume, cardiac output, and arterial-venous oxygen difference from rest to first threshold and those from rest to peak exercise were measured as the independent variables.

**Results:**

From rest to first threshold, the increases in arterial-venous oxygen difference (β = 0.711) and cardiac output (β = 0.572) were significant independent variables for the increase in $\dot{V}O_{2}$ (adjusted R^2^ = 0.877 p < 0.001). Similarly, from rest to peak exercise, the increases in arterial-venous oxygen difference (β = 0.665) and cardiac output (β = 0.636) were significant factors related to the increase in $\dot{V}O_{2}$ (adjusted R^2^ = 0.923, p < 0.001).

**Conclusion:**

Our results suggest that the ability of skeletal muscle to extract oxygen is a major cardiorespiratory factor related to the increase in $\dot{V}O_{2}$ during exercise testing in individuals with stroke. For improved cardiorespiratory fitness in individuals with stroke, the amount of functional muscle mass during exercise may need to be increased.

## Introduction

Individuals with stroke have reduced cardiorespiratory fitness compared with age- and sex-matched healthy individuals [1, 2]. Cardiorespiratory fitness reduction in individuals with stroke is potentially related to walking disability [3, 4], poor cognitive performance [5], and limitations in activities of daily living [6–8]. Low levels of cardiorespiratory fitness following stroke may lead to avoidance of physical activity, which causes further deconditioning [9, 10]. Therefore, understanding the cardiorespiratory factors related to cardiorespiratory fitness in individuals with stroke is essential for the development of appropriate therapies to improve physical activity levels and prevent further deconditioning.

From the physiological point of view, three phases and two thresholds can be defined with increasing exercise intensity [11]. Phase I is between rest and a first threshold. With increasing exercise intensity above the first threshold, the lactate production rate is higher than the metabolizing capacity of the muscle cell. Phase II is between the first threshold and a second threshold. Phase III is above the second threshold. With further increase in the workload above a second threshold, the muscular lactate production rate exceeds the systemic lactate elimination rate.

Oxygen consumption ($\dot{V}O_{2}$) at first threshold and that at peak exercise measured during a graded exercise test are used to assess cardiorespiratory fitness in individuals with stroke [1, 12–14]. The cardiorespiratory factors that potentially limit $\dot{V}O_{2}$ at peak exercise are respiratory and cardiac functions to supply oxygen, and the ability of skeletal muscles to extract oxygen [13, 15, 16]. In healthy adults, $\dot{V}O_{2}$ at peak exercise is limited by oxygen utilization among untrained individuals, while among trained individuals it is limited by oxygen supply [15–17]. Since the amount of active muscle mass determines whether the increase in $\dot{V}O_{2}$ during exercise is either centrally or peripherally limited (e.g. during exercise recruiting smaller muscle mass), the increase in $\dot{V}O_{2}$ may be limited by oxygen utilization, even in trained individuals [18]. The decrease in functional muscle mass due to paralysis may be one of the causes limiting cardiorespiratory responses to exercise [18]. Previous studies [13, 19–21] reported that tidal volume, heart rate, and arterial-venous oxygen difference at peak exercise are significantly lower in individuals with stroke than those in age- and sex-matched healthy adults, which may lead to the deterioration of cardiorespiratory fitness after stroke. Tomczak et al. [21] reported a significant difference between individuals with stroke and healthy adults in $\dot{V}O_{2}$ at peak exercise, but not in $\dot{V}O_{2}$ at rest. Thus, identifying the cardiorespiratory factors related to the increase in $\dot{V}O_{2}$ during exercise contributes to understanding the mechanisms of decrease in cardiorespiratory fitness in individuals with stroke. However, in individuals with stroke, the cardiorespiratory factors related to the increase in $\dot{V}O_{2}$ during exercise have not been examined using multivariate analysis.

Cross-sectional and longitudinal studies found a relationship between $\dot{V}O_{2}$ and arterial-venous oxygen difference at peak exercise in individuals with stroke [20, 22]. Therefore, we hypothesized that arterial-venous oxygen difference is a major cardiorespiratory factor related to the increase in $\dot{V}O_{2}$ during exercise in these individuals. In this study, we aimed to explore the cardiorespiratory factors related to the increase in $\dot{V}O_{2}$ from rest to first threshold and that from rest to peak exercise among individuals with stroke. The secondary aim was to determine the cardiorespiratory factors related to the increase in $\dot{V}O_{2}$ from first threshold to peak exercise, that from first threshold to second threshold, and that from second threshold to peak exercise in these individuals.

## Methods

### Study design

This study used a cross-sectional observational design. The study protocol was approved by the appropriate ethics committees of the Tokyo Bay Rehabilitation Hospital (approval number: 172–2) and the Shinshu University (approval number: 3813). All participants provided written informed consent prior to study enrollment. The study was conducted in accordance with the regulations of Declaration of Helsinki of 1964, as revised in 2013.

### Participants

Participants were recruited from a convalescent rehabilitation hospital between November 2017 and November 2018. The inclusion criteria for the study were as follows: (1) age 40–80 years, (2) being within 180 days after first-ever stroke, (3) ability to maintain a target cadence of 50 rpm during exercise, and (4) a Mini-Mental State Examination score [23] of 24 or more. The exclusion criteria were as follows: (1) limited range of motion and/or pain that could affect the exercise test, (2) unstable medical conditions such as unstable angina, uncontrolled hypertension, and tachycardia, (3) use of beta-blocker, and (4) any comorbid neurological disorder.

### Exercise testing

Participants were instructed to refrain from eating for 3 hours and to avoid caffeine and vigorous physical activity for at least 6 and 24 hours, respectively, before the exercise test [24]. All participants performed a symptom-limited graded exercise test on a recumbent cycle ergometer (Strength Ergo 240; Mitsubishi Electric Engineering Co., Ltd., Tokyo, Japan) that can be precisely load-controlled (coefficient of variation, 5%) over a wide range of pedaling resistance (0–400 W). The distance from the seat edge to pedal axis was adjusted so that the participant’s knee flexion angle was 20° when extended maximally. The backrest was set at 20° reclined from the vertical position. Additional strapping was attached to secure the paretic foot to the pedal as needed. Following a 3-min of rest period (in sitting position) on the recumbent cycle ergometer to establish a steady state, a warm-up was performed at 0 W for 3 min followed by 10 W increments every minute [24, 25]. Participants were instructed to maintain a target cadence of 50 rpm throughout the exercise [24, 25]. Blood pressure was monitored every minute from the non-paretic arm using an automated system (Tango; Sun Tech Medical Inc., NC, USA). The test was terminated if the participants showed signs of angina, dyspnea, inability to maintain cycling cadence more than 40 rpm, hypertension (more than 250 mmHg systolic or more than 115 mmHg diastolic), or a drop in systolic blood pressure of more than 10 mmHg despite an increase in work load [25, 26]. Participants provided their ratings of perceived exertion (6 = no exertion at all, 20 = maximal exertion) [27] for dyspnea and leg effort at the end of the test. Work rate at peak exercise was defined as the peak wattage on test termination [22]. To identify whether maximal effort was reached during the exercise test, at least 1 of the following criteria had to be met: (1) $\dot{V}O_{2}$ increased less than 150 mL·min^-1^ for more than 1 min despite increased work rate, (2) respiratory exchange ratio achieved greater than 1.10, (3) or heart rate achieved 85% of the age-predicted maximal heart rate (210 minus age) [28–30].

Participants rested for 5 min prior to obtaining the measurements. Cardiorespiratory variables were measured at rest for 3 min and continuously during exercise test. $\dot{V}O_{2}$, respiratory rate, tidal volume, and minute ventilation were measured on a breath-by-breath basis using an expired gas analyzer (Aerosonic AT-1100; ANIMA Corp., Tokyo, Japan). Carbon dioxide output, the ventilatory equivalents of oxygen and carbon dioxide, and the end-tidal oxygen and carbon dioxide fractions were also measured using the expired gas analyzer to determine first and second threshold. Heart rate, stroke volume, and cardiac output were measured on a beat-by-beat basis using a noninvasive impedance cardiography device (Task Force Monitor model 3040i; CN Systems Medizintechnik GmbH., Graz, Austria). The impedance cardiography method is a valid and reliable method for measuring cardiac hemodynamics at rest and during exercise [31–33]. The reproducibility of two consecutive measurements of stroke volume with the device was confirmed by the correlation coefficient, r = 0.971 [33]. The mean and standard deviations of the differences between two consecutive measurements of stroke volume are 0.845 ± 2.549 mL [33]. Measurement values of cardiorespiratory variables were interpolated to 1-s intervals, time-aligned, and averaged into 5-s bins. This approach was used to assess the cardiorespiratory responses to exercise in healthy adults [34] and in individuals with stroke [21]. We could derive arterial-venous oxygen difference on a second-by-second basis by converting the breath-by-breath expired gas data and the beat-by-beat cardiac hemodynamics data into second-by-second data. Arterial-venous oxygen difference was calculated as the ratio between $\dot{V}O_{2}$ and cardiac output according to the Fick’s equation: $\dot{V}O_{2}$ = cardiac output × arterial-venous oxygen difference [35].

Cardiorespiratory variables at rest were defined as the average value obtained during 1 min before exercise onset, and those at peak exercise were defined as the average value obtained during the last 30 s of exercise test [21, 24]. The first threshold was determined using a combination of the following criteria: (1) the time point where the ventilatory equivalent of oxygen reached its minimum or started to increase, without an increase in the ventilatory equivalent of carbon dioxide; (2) the time point at which the end-tidal oxygen fraction reached a minimum or started to increase, without a decline in the end-tidal carbon dioxide fraction; and (3) the time point of deflection of carbon dioxide output versus $\dot{V}O_{2}$ [11]. The first two methods were prioritized in case the three methods presented different results [36, 37]. The second threshold is the intensity at which the muscular lactate production rate begins to exceed the systemic lactate elimination rate [11]. The second threshold was determined by: (1) the minimal value or nonlinear increase in the ventilatory equivalent of carbon dioxide; (2) the time point at which the end-tidal carbon dioxide fraction started to decline; and (3) the time point of deflection of minute ventilation versus $\dot{V}O_{2}$ [11]. The first two criteria were prioritized in case the three methods presented different results [36]. The first and second threshold were determined as an average based on the values provided by two independent raters (NI and YS), when the difference in $\dot{V}O_{2}$ values of the corresponding points as determined by the two raters was less than 100 mL·min^-1^ [37, 38]. In case of any discrepancy, a third experienced rater (KO) judged the time point, and either the first or second threshold was used as the average of the two closest values [36, 37].

### Functional impairment assessment

A lower extremity motor subscale of Fugle-Meyer Assessment [39] was used to assess functional impairment in the paretic lower extremity. The possible score ranged from 0 to 34 points.

### Statistical analysis

The G Power computer program version 3.1.9.2 (Heinrich Heine University, Dusseldorf, Germany) [40] was used to calculate the sample size required for multiple regression analysis. If up to seven variables (respiratory rate, tidal volume, minute ventilation, heart rate, stroke volume, cardiac output, and arterial-venous oxygen difference) would be modeled at an effect size of 0.49 (very large), α level of 0.05 and power of 0.80, a minimum of 13 participants would be required [40, 41].

Normality of distribution was tested using the Shapiro Wilk test. One-way repeated-measures analysis of variance or Friedman test with exercise period as a factor was used to examine whether cardiorespiratory variables changed during exercise. Post hoc analyses were performed using the Bonferroni multiple comparison test.

The increase in $\dot{V}O_{2}$ from rest to first threshold and that from rest to peak exercise were calculated as the dependent variables. The increase in $\dot{V}O_{2}$ from first threshold to peak exercise, that from first threshold to second threshold, and that from second threshold to peak exercise were also calculated as the dependent variables. Pearson’s product moment correlation coefficient or Spearman’s rank correlation coefficient was used to test the correlations between the increases in $\dot{V}O_{2}$ and other cardiorespiratory variables. Pearson’s product moment correlation coefficient or Spearman’s rank correlation coefficient was also used to examine if age, functional impairment, and other anthropometric characteristics including height, body mass, and body mass index were related to the increase in $\dot{V}O_{2}$ during exercise testing [25, 26, 37, 42]. We performed these correlation analyses to identify independent variables that were entered in the stepwise multiple regression analysis. Variables that significantly correlated with the increase in $\dot{V}O_{2}$ during exercise testing were then entered in the stepwise multiple regression analysis to identify the cardiorespiratory factors related to the increase in $\dot{V}O_{2}$, while considering multicollinearity. When age, functional impairment, and/or anthropometric characteristics were selected as independent variables for multiple regression analysis, we found potentially confounding effects of age, functional impairment, and/or anthropometric characteristics on the relationships between the increases in $\dot{V}O_{2}$ and other cardiorespiratory variables during exercise testing. Statistical analyses were performed using the Statistical Package for the Social Sciences software version 24.0 (International Business Machines Corp., NY, USA). Any p values less than 0.05 were considered statistically significant.

## Results

**Fig 1:**
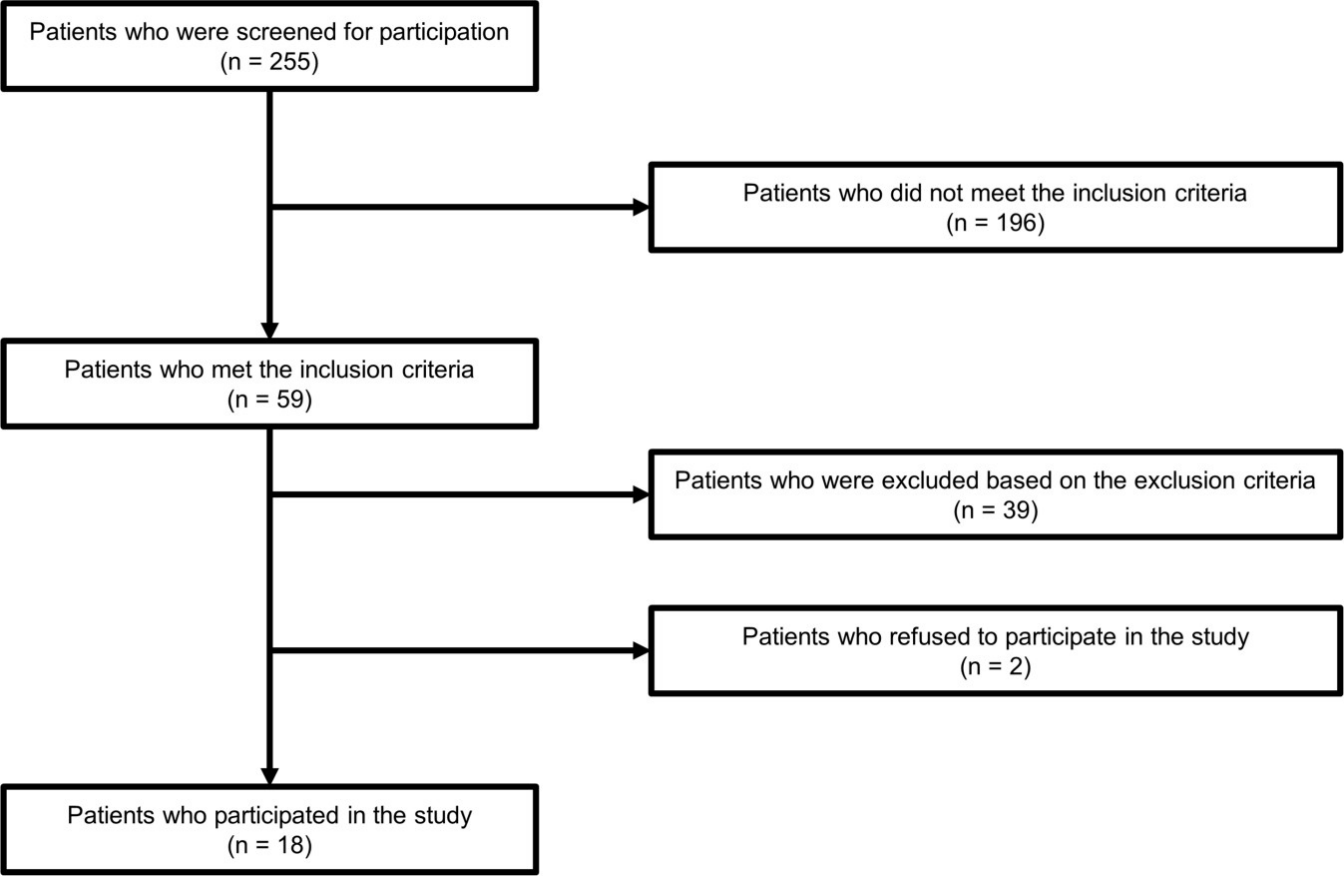
Flow diagram of study participants.

A flow diagram of study participants is shown in Fig 1. Eighteen individuals with stroke participated in the study. Table 1 shows the characteristics of the participants.

**Table 1:** Characteristics of participants.

Variable	Value
Age, years	60.1 ± 9.4
Sex, male/female	14 (78)/4 (22)
Height, m	1.66 ± 0.07
Body mass, kg	63.6 ± 8.9
Body mass index, kg·m^-2^	23.0 ± 3.3
Type of stroke, ischemic/hemorrhage	11 (61)/7 (39)
Side of motor paresis, right/left	8 (44)/10 (56)
Time since stroke, days	67.1 ± 30.8
Fugl-Meyer lower extremity motor scores, points	28.8 ± 7.7
Antihypertensive medications	
Angiotensin II receptor blocker	2 (11)
Calcium channel blocker	4 (22)
Comorbidities	
Hypertension	6 (33)
Diabetes mellitus	4 (22)
Hyperlipidemia	3 (17)

Values are presented as mean ± SD or number (%).

No significant adverse events occurred during or after the exercise test. All participants stopped the exercise test due to their inability to maintain cycling cadence more than 40 rpm. With respect to each of the 3 criteria for reaching maximal effort, 16 participants (89%) showed the increase in $\dot{V}O_{2}$ less than 150 mL·min^-1^ for more than 1 min despite increased work rate, 4 participants (22%) achieved a respiratory exchange ratio value greater than 1.10, and 9 participants (50%) reached 85% of the age-predicted maximal heart rate. All participants met at least one of the criteria for reaching maximal effort. One and nine participants met three and two criteria for reaching maximal effort, respectively. Median (interquartile range) values of the ratings of perceived exertion for dyspnea and leg effort at the end of the test were 13 (13–15) and 15 (13–15), respectively. Mean ± standard deviation of respiratory exchange ratio and work rate at peak exercise were 0.98 ± 0.13 and 69.4 ± 30.6 W, respectively.

**Fig 2:**
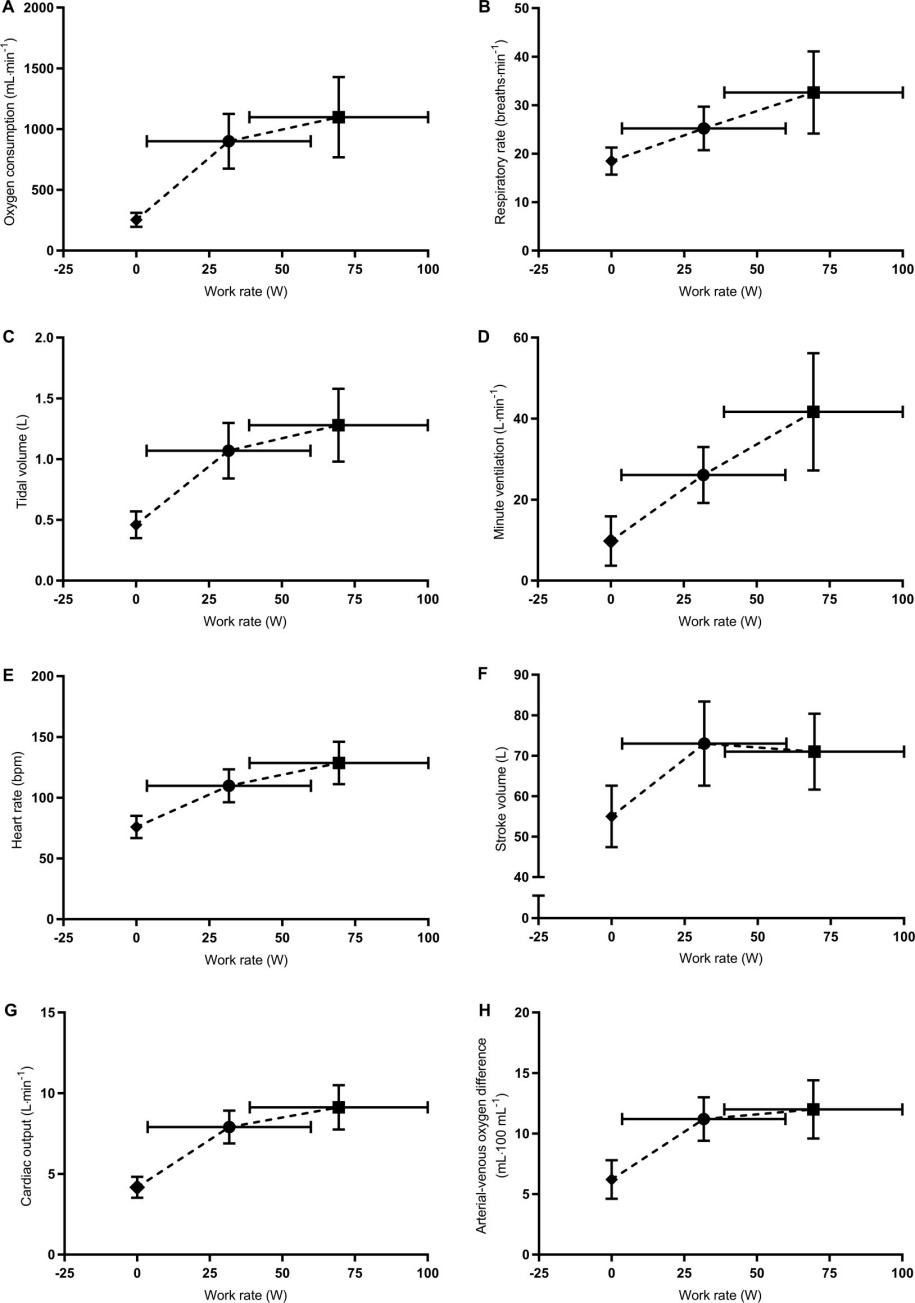
Relationships of work rate with $\dot{V}O_{2}$ (A), respiratory rate (B), tidal volume (C), minute ventilation (D), heart rate (E), stroke volume (F), cardiac output (G), and arterial-venous oxygen difference (H) during the exercise test. Diamonds, circles, and squares represent the mean values at rest, first threshold, and peak exercise, respectively. Vertical and horizontal bars represent standard deviation. The data of second threshold were excluded, as it was obtained from only 11 participants.

Measurement values at rest, first threshold, second threshold, and peak exercise are shown in Table 2 and Fig 2. The first threshold was determined in all participants, while the second threshold could be determined in only 11/18 participants. Therefore, we excluded the data of second threshold from statistical analyses. We observed a main effect of exercise period on all cardiorespiratory variables (p < 0.001). All cardiorespiratory variables at first threshold were significantly higher than those at rest (p < 0.001). From first threshold to peak exercise, cardiorespiratory variables, except for stroke volume and arterial-venous oxygen difference increased significantly (p < 0.001).

**Table 2:** Cardiorespiratory variables at rest, first threshold, second threshold, and peak exercise.

Variable	Rest	First threshold	Second threshold (n = 11)	Peak exercise	p value
$\dot{V}O_{2}$, mL·min^-1^	253.1 ± 57.7	899.7 ± 224.8*	972.1 ± 224.2	1098.3 ± 330.3^†^^,^ ^‡^	< 0.001
Respiratory rate, breaths·min^-1^	18.5 ± 2.8	25.2 ± 4.5*	25.8 ± 3.9	32.6 ± 8.5^†^^,^ ^‡^	< 0.001
Tidal volume, L	0.46 ± 0.11	1.07 ± 0.23*	1.17 ± 0.19	1.28 ± 0.30^†^^,^ ^‡^	< 0.001
Minute ventilation, L·min^-1^	8.5 ± 1.8	26.1 ± 6.9*	30.4 ± 8.1	41.7 ± 14.5^†^^,^ ^‡^	< 0.001
Heart rate, bpm	76.0 ± 9.2	109.9 ± 13.6*	114.2 ± 12.1	128.6 ± 17.5^†^^,^ ^‡^	< 0.001
Stroke volume, mL	55.0 ± 7.6	73.0 ± 10.4*	74.6 ± 10.3	71.0 ± 9.4^†^	< 0.001
Cardiac output, L·min^-1^	4.17 ± 0.65	7.91 ± 1.02*	8.44 ± 1.00	9.12 ± 1.38^†^^,^ ^‡^	< 0.001
Arterial-venous oxygen difference, mL·100 mL^-1^	6.2 ± 1.6	11.2 ± 1.8*	11.5 ± 1.9	12.0 ± 2.4^†^	< 0.001
Respiratory exchange ratio	0.74 ± 0.06	0.79 ± 0.06*	0.87 ± 0.10	0.98 ± 0.13^†^^,^ ^‡^	< 0.001
Rating of perceived exertion for dyspnea	NA	NA	NA	13 (13–15)	NA
Rating of perceived exertion for leg effort	NA	NA	NA	15 (13–15)	NA
Work rate, W	0 ± 0	31.7 ± 28.1	47.3 ± 34.1	69.4 ± 30.6	NA

Values are presented as mean ± SD or median (interquartile range).

The data of second threshold were excluded from the statistical analyses.

p value represents a significant main effect of the exercise period.

*, a significant difference between first threshold and rest (p < 0.05).

^†^, a significant difference between peak exercise and rest (p < 0.05).

^‡^, a significant difference between peak exercise and first threshold (p < 0.05).

NA, not applicable.

**Fig 3:**
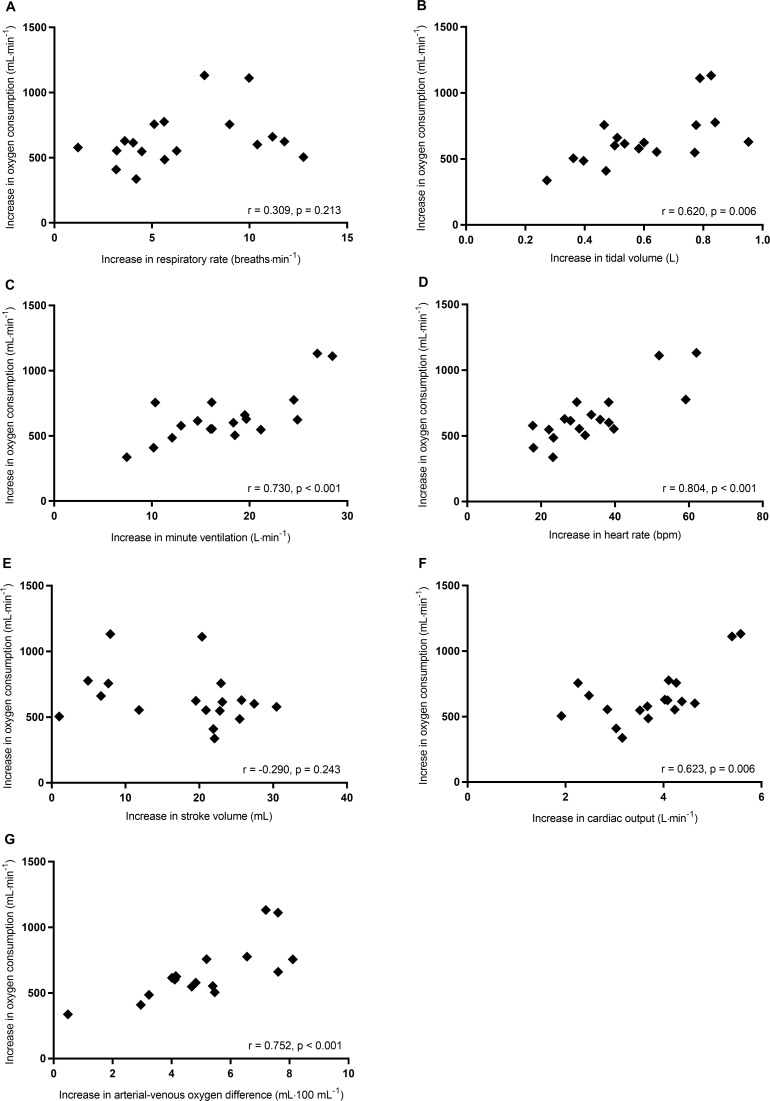
Correlations of the increases in $\dot{V}O_{2}$ with respiratory rate (A), tidal volume (B), minute ventilation (C), heart rate (D), stroke volume (E), cardiac output (F), and arterial-venous oxygen difference (G) from rest to first threshold.

From rest to first threshold, correlations between the increases in $\dot{V}O_{2}$ and other cardiorespiratory variables are shown in Table 3 and Fig 3. The increase in $\dot{V}O_{2}$ significantly correlated with the increases in tidal volume (Fig 3B), minute ventilation (Fig 3C), heart rate (Fig 3D), cardiac output (Fig 3F), and arterial-venous oxygen difference (Fig 3G). The increases in $\dot{V}O_{2}$ did not significantly correlate with age, Fugl-Meyer lower extremity motor scores, and anthropometric characteristics (Table 3). Stepwise multiple regression analysis revealed that the increases in arterial-venous oxygen difference (β = 0.711) and cardiac output (β = 0.572) were the significant independent variables for the increase in $\dot{V}O_{2}$ (adjusted R^2^ = 0.877, p < 0.001) (Table 4). The increase in arterial-venous oxygen difference was a major cardiorespiratory factor related to the increase in $\dot{V}O_{2}$ from rest to first threshold.

**Table 3:** Correlations between the increases in $\dot{V}O_{2}$ from rest to first threshold and the increases in other cardiorespiratory variables, age, functional impairment, and anthropometric characteristics.

Variable	Increase in $\dot{V}O_{2}$		
	r	95% CI	p value
Increase in respiratory rate	0.309	-0.185, 0.678	0.213
Increase in tidal volume	0.620	0.215, 0.843	0.006
Increase in minute ventilation	0.730	0.399, 0.893	< 0.001
Increase in heart rate	0.804	0.540, 0.924	< 0.001
Increase in stroke volume	-0.290	-0.667, 0.204	0.243
Increase in cardiac output	0.623	0.221, 0.845	0.006
Increase in arterial-venous oxygen difference	0.752	0.440, 0.902	< 0.001
Age	-0.055	-0.509, 0.423	0.829
Fugl-Meyer lower extremity motor scores	0.168	-0.325, 0.589	0.506
Height	0.080	-0.402, 0.527	0.752
Body mass	0.302	-0.192, 0.674	0.224
Body mass index	0.221	-0.275, 0.623	0.379

r, correlation coefficient; 95% CI, 95% confidence interval

**Table 4:** Stepwise multiple regression analysis for identifying factors related to the increases in $\dot{V}O_{2}$ from rest to first threshold.

Variable	β	Coefficient	SE	t value	p value
Increase in arterial-venous oxygen difference from rest to first threshold	0.711	76.91	9.24	8.32	< 0.001
Increase in cardiac output from rest to first threshold	0.572	118.32	17.69	6.68	< 0.001
Constant		-184.99	80.01	-2.31	0.035
F (2, 15) = 61.37, p < 0.001, R^2^ = 0.891, Adjusted R^2^ = 0.877					

β, standard coefficient; SE, standard error

**Fig 4:**
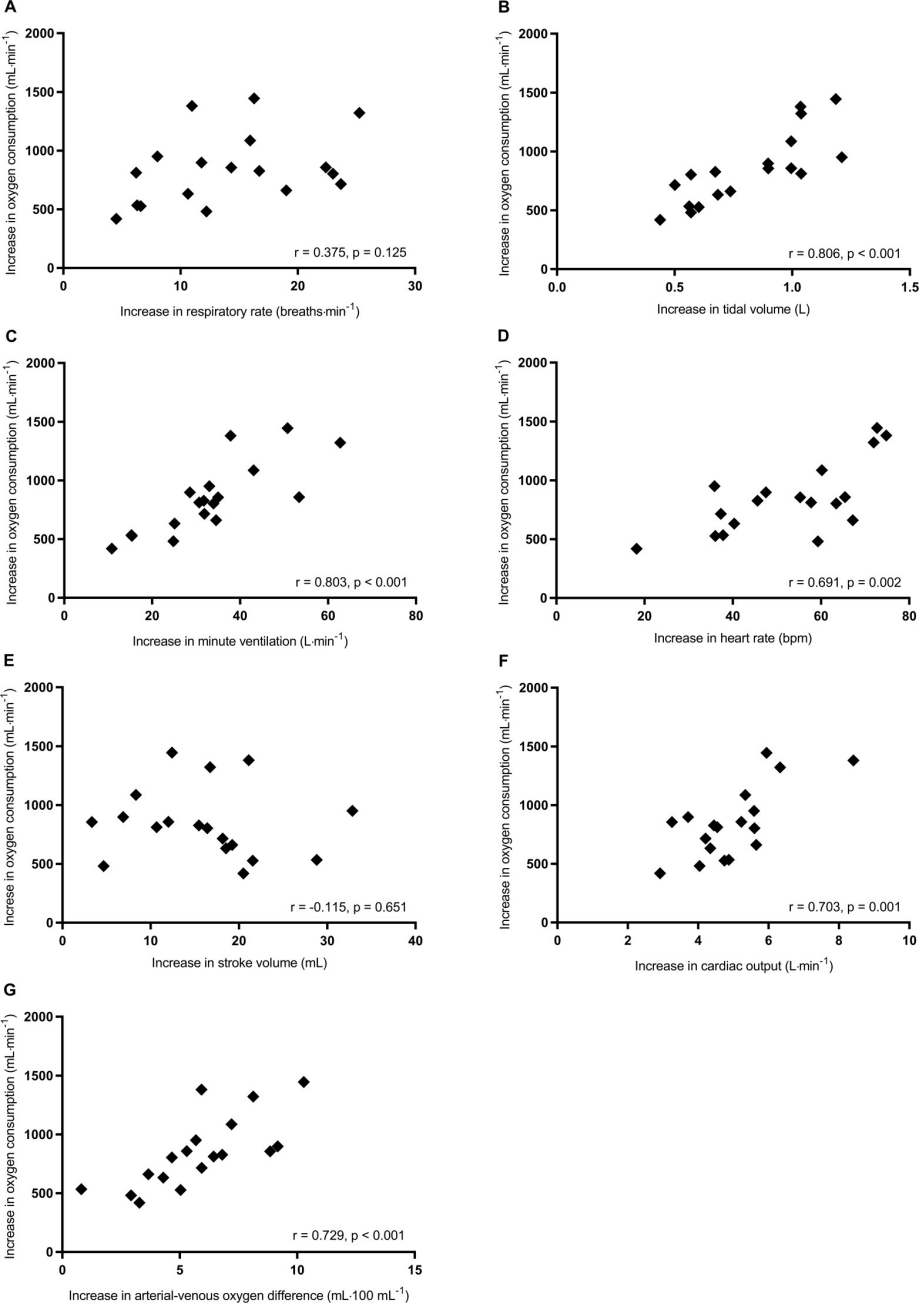
Correlations of the increases in $\dot{V}O_{2}$ with respiratory rate (A), tidal volume (B), minute ventilation (C), heart rate (D), stroke volume (E), cardiac output (F), and arterial-venous oxygen difference (G) from rest to peak exercise.

From rest to peak exercise, correlations between the increases in $\dot{V}O_{2}$ and other cardiorespiratory variables are shown in Table 5 and Fig 4. The increases in $\dot{V}O_{2}$ significantly correlated with the increases in tidal volume (Fig 4B), minute ventilation (Fig 4C), heart rate (Fig 4D), cardiac output (Fig 4F), and arterial-venous oxygen difference (Fig 4G). The increases in $\dot{V}O_{2}$ also significantly correlated with body mass (Table 5). Stepwise multiple regression analysis revealed that the increases in arterial-venous oxygen difference (β = 0.665) and cardiac output (β = 0.636) were significant factors related to the increase in $\dot{V}O_{2}$ (adjusted R^2^ = 0.923, p < 0.001) (Table 6). The increase in arterial-venous oxygen difference was a major cardiorespiratory factor related to the increase in $\dot{V}O_{2}$ from rest to peak exercise.

**Table 5:** Correlations between the increase in $\dot{V}O_{2}$ from rest to peak exercise and the increases in other cardiorespiratory variables, age, functional impairment, and anthropometric characteristics.

Variable	Increase in $\dot{V}O_{2}$		
	r	95% CI	p value
Increase in respiratory rate	0.375	-0.111, 0.717	0.125
Increase in tidal volume	0.806	0.544, 0.925	< 0.001
Increase in minute ventilation	0.803	0.537, 0.923	< 0.001
Increase in heart rate	0.691	0.330, 0.875	0.002
Increase in stroke volume	-0.115	-0.552, 0.372	0.651
Increase in cardiac output	0.703	0.352, 0.881	0.001
Increase in arterial-venous oxygen difference	0.729	0.398, 0.892	< 0.001
Age	-0.094	-0.537, 0.390	0.710
Fugl-Meyer lower extremity motor scores	0.423	-0.055, 0.743	0.080
Height	0.309	-0.184, 0.678	0.212
Body mass	0.554	0.117, 0.811	0.017
Body mass index	0.349	-0.141, 0.701	0.156

r, correlation coefficient; 95% CI, 95% confidence interval

**Table 6:** Stepwise multiple regression analysis for identifying factors related to the increases in $\dot{V}O_{2}$ from rest to peak exercise.

Variable	β	Coefficient	SE	t value	p value
Increase in arterial-venous oxygen difference from rest to peak exercise	0.665	83.85	8.54	9.82	< 0.001
Increase in cardiac output from rest to peak exercise	0.636	152.22	16.22	9.38	< 0.001
Constant		-395.25	92.20	-4.29	0.001
F (2, 15) = 102.57, p < 0.001, R^2^ = 0.932, Adjusted R^2^ = 0.923					

β, standard coefficient: SE, standard error

**Fig 5:**
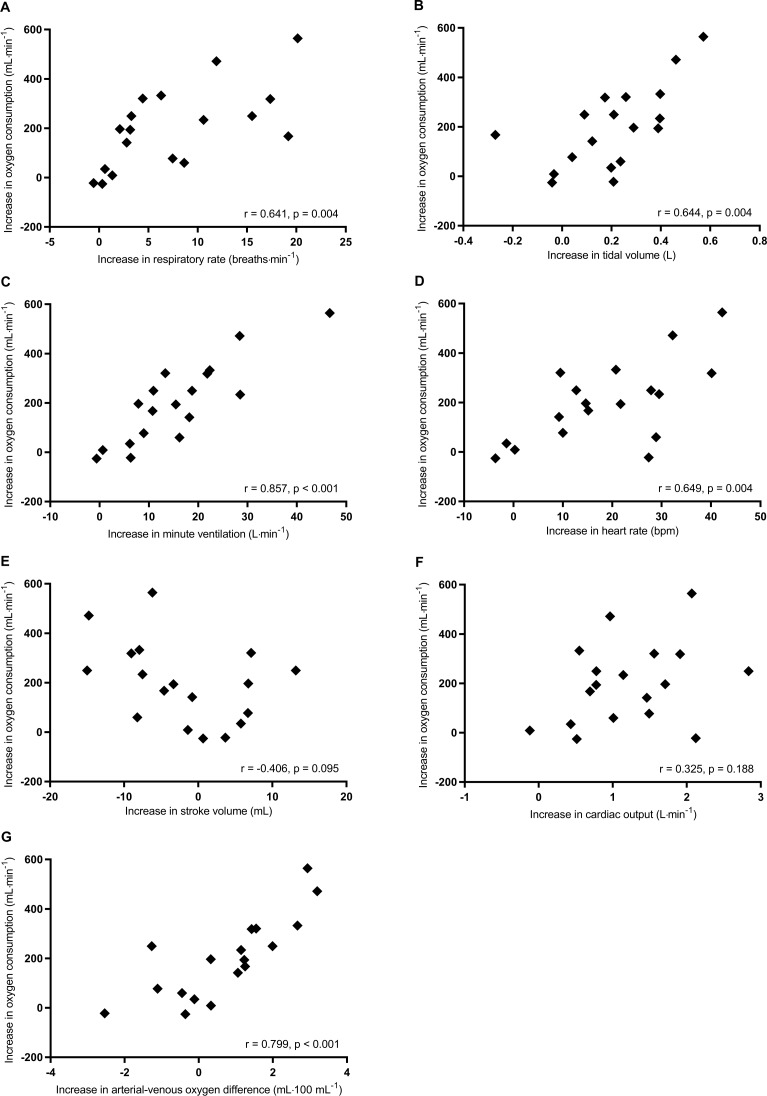
Correlations of the increases in $\dot{V}O_{2}$ with respiratory rate (A), tidal volume (B), minute ventilation (C), heart rate (D), stroke volume (E), cardiac output (F), and arterial-venous oxygen difference (G) from first threshold to peak exercise.

From first threshold to peak exercise, correlations between the increases in $\dot{V}O_{2}$ and other cardiorespiratory variables are shown in Table 7 and Fig 5. The increases in $\dot{V}O_{2}$ significantly correlated with the increases in respiratory rate (Fig 5A), tidal volume (Fig 5B), minute ventilation (Fig 5C), heart rate (Fig 5D), and arterial-venous oxygen difference (Fig 5G). The increases in $\dot{V}O_{2}$ also significantly correlated with Fugl-Meyer lower extremity motor scores and body mass (Table 7). Stepwise multiple regression analysis revealed that the increases in minute ventilation (β = 0.584) and arterial-venous oxygen difference (β = 0.389) were significant independent variables associated with for the increase in $\dot{V}O_{2}$ (adjusted R^2^ = 0.786, p < 0.001) (Table 8).

**Table 7:** Correlations between the increases in $\dot{V}O_{2}$ from first threshold to peak exercise and the increases in other cardiorespiratory variables, age, functional impairment, and anthropometric characteristics.

Variable	Increase in $\dot{V}O_{2}$		
	r	95% CI	p value
Increase in respiratory rate	0.641	0.249, 0.853	0.004
Increase in tidal volume	0.644	0.253, 0.854	0.004
Increase in minute ventilation	0.857	0.651, 0.946	< 0.001
Increase in heart rate	0.649	0.261, 0.856	0.004
Increase in stroke volume	-0.406	-0.734, 0.075	0.095
Increase in cardiac output	0.325	-0.167, 0.688	0.188
Increase in arterial-venous oxygen difference	0.799	0.531, 0.922	< 0.001
Age	-0.104	-0.545, 0.381	0.681
Fugl-Meyer lower extremity motor scores	0.566	0.135, 0.817	0.014
Height	0.468	0.001, 0.767	0.050
Body mass	0.638	0.244, 0.851	0.004
Body mass index	0.363	-0.125, 0.710	0.138

r, correlation coefficient; 95% CI, 95% confidence interval

**Table 8:** Stepwise multiple regression analysis for identifying factors related to the increases in $\dot{V}O_{2}$ from first threshold to peak exercise.

Variable	β	Coefficient	SE	t value	p value
Increase in minute ventilation from first threshold to peak exercise	0.584	8.436	2.28	3.70	0.002
Increase in arterial-venous oxygen difference from first threshold to peak exercise	0.389	41.78	16.98	2.46	0.027
Constant		36.25	33.45	1.08	0.296
F (2, 15) = 32.22, p < 0.001, R^2^ = 0.811, Adjusted R^2^ = 0.786					

β, standard coefficient; SE, standard error

The cardiorespiratory factors related to the increase in $\dot{V}O_{2}$ from first threshold to second threshold and then from second threshold to peak exercise were determined in 11 participants who reached second threshold. The increase in $\dot{V}O_{2}$ from first threshold to second threshold was not correlated with the increases in other cardiorespiratory variables (Table 9). From second threshold to peak exercise, the increases in $\dot{V}O_{2}$ significantly correlated with the increases in respiratory rate, tidal volume, minute ventilation, heart rate, and arterial-venous oxygen difference (Table 10). The increases in $\dot{V}O_{2}$ did not significantly correlate with age, Fugl-Meyer lower extremity motor scores, and anthropometric characteristics (Table 10). Stepwise multiple regression analysis revealed that the increase in minute ventilation (β = 0.850) was a significant independent variable associated with the increase in $\dot{V}O_{2}$ (adjusted R^2^ = 0.691, p = 0.001) (Table 11).

**Table 9:** Correlations between the increases in $\dot{V}O_{2}$ from first threshold to second threshold and the increases in other cardiorespiratory variables, age, functional impairment, and anthropometric characteristics (n = 11).

Variable	Increase in $\dot{V}O_{2}$		
	r	95% CI	p value
Increase in respiratory rate	-0.050	-0.631, 0.567	0.885
Increase in tidal volume	0.598	-0.003, 0.882	0.052
Increase in minute ventilation	0.601	0.001, 0.883	0.051
Increase in heart rate	0.485	-0.162, 0.841	0.130
Increase in stroke volume	-0.155	-0.691, 0.491	0.650
Increase in cardiac output	0.156	-0.490, 0.691	0.647
Increase in arterial-venous oxygen difference	0.487	-0.159, 0.841	0.129
Age	0.476	-0.174, 0.837	0.139
Fugl-Meyer lower extremity motor scores	0.240	-0.421, 0.734	0.477
Height	-0.005	-0.603, 0.597	0.989
Body mass	0.092	-0.537, 0.656	0.787
Body mass index	0.053	-0.565, 0.633	0.876

r, correlation coefficient; 95% CI, 95% confidence interval

**Table 10:** Correlations between the increases in $\dot{V}O_{2}$ from second threshold to peak exercise and the increases in other cardiorespiratory variables, age, functional impairment, and anthropometric characteristics (n = 11).

Variable	Increase in $\dot{V}O_{2}$		
	r	95% CI	p value
Increase in respiratory rate	0.747	0.266, 0.930	0.008
Increase in tidal volume	0.823	0.441, 0.953	0.002
Increase in minute ventilation	0.850	0.509, 0.960	0.001
Increase in heart rate	0.803	0.391, 0.947	0.003
Increase in stroke volume	-0.246	-0.737, 0.415	0.466
Increase in cardiac output	0.559	-0.006, 0.868	0.074
Increase in arterial-venous oxygen difference	0.689	0.152, 0.912	0.019
Age	-0.225	-0.727, 0.434	0.507
Fugl-Meyer lower extremity motor scores	0.568	-0.048, 0.871	0.068
Height	0.360	-0.306, 0.790	0.276
Body mass	0.433	-0.226, 0.820	0.184
Body mass index	0.115	-0.521, 0.669	0.735

r, correlation coefficient; 95% CI, 95% confidence interval

**Table 11:** Stepwise multiple regression analysis for identifying factors related to the increases in $\dot{V}O_{2}$ from second threshold to peak exercise (n = 11).

Variable	β	Coefficient	SE	t value	p value
Increase in minute ventilation from second threshold to peak exercise	0.850	12.46	2.58	4.83	0.001
Constant		-10.95	49.06	-0.22	0.828
F (1, 9) = 23.35, p = 0.001, R^2^ = 0.722, Adjusted R^2^ = 0.691					

β, standard coefficient; SE, standard error

## Discussion

This is the first study to explore cardiorespiratory factors related to the increase in $\dot{V}O_{2}$ during graded exercise in individuals with stroke. This study demonstrated that the increase in arterial-venous oxygen difference was a major cardiorespiratory factor related to both the increases in $\dot{V}O_{2}$ from rest to first threshold and that from rest to peak exercise. Our results also demonstrated no significant confounding effects of age, functional impairment, and anthropometric characteristics on the relationships between increases in $\dot{V}O_{2}$ and other cardiorespiratory variables during exercise testing. These findings suggest that the impaired ability of skeletal muscles to extract oxygen is a main cardiorespiratory factor related to the decrease in cardiorespiratory fitness in individuals with stroke.

The decrease in functional muscle mass due to paralysis can limit the increases in $\dot{V}O_{2}$ and other cardiorespiratory variables during exercise testing. The influences of the amount of active muscle mass on cardiorespiratory responses to exercise have been investigated by comparing cardiorespiratory outcomes during one-legged and two-legged cycling exercises in healthy people [18]. $\dot{V}O_{2}$ at first threshold and that at peak exercise are lower during one-legged cycling exercises compared to those during two-legged cycling exercises [43–50]. Minute ventilation and arterial-venous oxygen difference responses are also lower during one-legged cycling exercises [45, 46, 51]. Furthermore, the level of catecholamines is lower during one-legged cycling exercises compared to that in two-legged cycling exercises [46, 52]. As catecholamines stimulate cardiorespiratory functions, lower levels of catecholamines during one-legged cycle exercises explain the lower cardiorespiratory responses [18]. Although we did not assess the amount of functional muscle mass during exercise, the above studies suggest that the decrease in functional muscle mass due to paralysis can explain the relationships between increases in $\dot{V}O_{2}$ and other cardiorespiratory variables during exercise testing observed in this study.

The increase in arterial-venous oxygen difference was a major independent variable for the increases in $\dot{V}O_{2}$ from rest to first threshold and that from rest to peak exercise. From first threshold to peak exercise, the increase in arterial-venous oxygen difference was also an independent variable for the increases in $\dot{V}O_{2}$, while cardiac output was not. These results support the findings of Jakovljevic et al. [20] and Moore et al. [22] who reported that oxygen extraction rather than oxygen supply is related with cardiorespiratory fitness in individuals with stroke. Skeletal muscle changes after stroke, such as muscle atrophy and shift of muscle fiber type (from type I slow-twitch muscle fibers to type II fast-twitch muscle fibers) particularly in the paretic lower extremity, are observed in individuals with stroke [53]. The impaired vasodilatory function and reduction in blood flow in the paretic lower extremity have also been reported [54, 55]. In addition to the decrease in functional muscle mass during exercise, these changes in skeletal muscles after stroke can reduce their ability to extract oxygen. This may further increase the dependence on anaerobic glycolysis for energy output, thus increasing the output of lactate [56, 57]. However, from our respiratory exchange ratio data, we expected blood lactate concentration to remain low in this study. These findings support the relationships between the increases in $\dot{V}O_{2}$ and arterial-venous oxygen difference during exercise testing observed in this study.

Furthermore, this study demonstrated that the increase in cardiac output was related to the increases in $\dot{V}O_{2}$ from rest to first threshold and that from rest to peak exercise irrespective of the increase in arterial-venous oxygen difference. Tomczak et al. [21] reported that the impaired increase in $\dot{V}O_{2}$ during exercise testing in individuals with stroke is attributed to the impaired increase in cardiac output, which in turn could be attributed to the impaired increase in heart rate. From first threshold to peak exercise, we observed the significant increases in heart rate and cardiac output, but not in stroke volume. These results suggest that the increase in heart rate contributed to the increase in cardiac output in this phase. In addition, our correlational analysis indicated that the increase in $\dot{V}O_{2}$ during exercise testing was related with the increase in heart rate, but not the increase in stroke volume. These results of our study support the findings of Tomczak et al. [21]. As mentioned above, the increases in heart rate and cardiac output during exercise testing may be limited by lower levels of catecholamines due to the decrease in functional muscle mass after stroke [18]. In addition, the decreased functional muscle mass in individuals with stroke can reduce the increases in heart rate and cardiac output, just matching the needs of the lower muscle mass [18]. These findings can explain the relationship between the increases in $\dot{V}O_{2}$ and cardiac output during exercise testing observed in this study.

Sisante et al. [19] and Tomczak et al. [21] reported that tidal volume, minute ventilation, and $\dot{V}O_{2}$ at peak exercise were significantly lower in the stroke group than in control, while there was no significant difference in respiratory rate at peak exercise between the groups. Therefore, the decrease in tidal volume is believed to limit minute ventilation and $\dot{V}O_{2}$ at peak exercise in individuals with stroke [19, 21]. The paralysis of expiratory muscles on the affected side, decreased motion of the diaphragm, and reduced chest wall excursion may limit the increases in tidal volume during exercise [13, 58]. These findings support the relationship between increases in $\dot{V}O_{2}$ and the increases in tidal volume and minute ventilation during exercise testing. Both from rest to first threshold and from rest to peak exercise, there was no significant correlation between the increases in $\dot{V}O_{2}$ and respiratory rate. Therefore, the tidal volume response may be related to $\dot{V}O_{2}$ response irrespective of respiratory rate response during exercise testing. The increase in minute ventilation was a major independent variable for the increase in $\dot{V}O_{2}$ from first threshold to peak exercise, and then from second threshold to peak exercise, but not from rest to peak exercise. There was no occurrence of cardiorespiratory factors related to the increase in $\dot{V}O_{2}$ from first threshold to second threshold, which may be attributed to low increment of $\dot{V}O_{2}$ in this phase. The increment of $\dot{V}O_{2}$ from rest to first threshold accounted for approximately 75% of the increment of $\dot{V}O_{2}$ from rest to peak exercise. This may explain why the increases in arterial-venous oxygen difference and cardiac output rather than the increase in minute ventilation were selected as the independent variables for the increases in $\dot{V}O_{2}$ from rest to peak exercise. These results suggest that the ability of skeletal muscles to extract oxygen and cardiac function rather than respiratory function are related to cardiorespiratory fitness in individuals with stroke.

Considering the influences of the amount of active muscle mass on cardiorespiratory responses to exercise [18], it is important to increase the amount of functional muscle mass during exercise for enhancing the cardiorespiratory responses in individuals with stroke. Therefore, exercises that recruit more muscle mass, such as combined arm and leg exercises could be beneficial to improve cardiorespiratory fitness in these individuals.

Studies reported that functional impairment is related to cardiorespiratory responses during exercise testing in individuals with stroke [25, 37, 59]. However, we found no significant confounding effects of functional impairment on the relationships between the increases in $\dot{V}O_{2}$ and other cardiorespiratory variables during exercise testing, which may be attributed to the fact that our study participants presented with relatively mild functional impairment. Although all participants stopped the exercise test due to their inability to maintain cycling cadence, seven participants did not reach the second threshold during exercise testing. In addition, 14 participants did not achieve a respiratory exchange ratio value greater than 1.10. The relatively low ratings of perceived exertion at the end of the test may be explained by the low number of participants who reached the second threshold and/or respiratory exchange ratio value greater than 1.10. Although a respiratory exchange ratio greater than 1.10 is generally considered an indication of excellent subject effort during exercise testing [35], as several studies also reported, individuals with hemiparetic stroke find it difficult to reach the respiratory exchange ratio greater than 1.10 during exercise testing on a recumbent cycle ergometer [24, 37, 60]. In individuals with stroke, impairments in strength, coordination, muscle endurance, and sensorimotor control contribute to difficulties in pedaling at a high work rate [60]. The $\dot{V}O_{2}$ reserve and heart rate reserve percentages were recommended for prescribing aerobic exercise intensity for individuals with stroke [61]. Therefore, it is probably difficult for individuals with stroke to achieve sufficient intensity of exercise prescription during exercise testing using a recumbent cycle ergometer. An exercise test, using the combined arm and leg modality such as the total-body recumbent stepper, may be useful for guiding exercise prescription in these individuals [42].

This study had several limitations. First, all participants were in the subacute stages of recovery from stroke. Therefore, generalization of the findings to individuals with chronic stroke should be made with caution. Second, the sample size was relatively small, although that was determined based on power analysis. Therefore, we could not perform subgroup analyses to determine whether cardiorespiratory variables related to the increase in $\dot{V}O_{2}$ during exercise testing would be different between participants who reached and those who failed to reach the second threshold or participants who reached and those who failed to reach a respiratory exchange ratio value greater than 1.10. Third, we used a recumbent cycle ergometer. A treadmill [6], a total-body recumbent stepper [42], a robotics-assisted tilt table [36], and an arm crank ergometer [37] are also used to assess cardiorespiratory fitness in individuals with stroke. Differences in the amount of active muscle mass among exercise devices may affect the relationships observed between the increases in $\dot{V}O_{2}$ and other cardiorespiratory variables during exercise testing. Further studies are warranted to examine whether the major cardiorespiratory factors related to the increase in $\dot{V}O_{2}$ during exercise differ among different exercise devices. Finally, as this study used a cross-sectional observational design, the factors related to the temporal changes in $\dot{V}O_{2}$ at first threshold and at peak exercise could not be examined. Further longitudinal studies are needed to examine whether impairments in arterial-venous oxygen difference and cardiac output affect the temporal changes in $\dot{V}O_{2}$ for the development of appropriate therapies to improve cardiorespiratory fitness in individuals with stroke.

## Conclusions

Our results suggest that the ability of skeletal muscles to extract oxygen is a major cardiorespiratory factor related to the increase in $\dot{V}O_{2}$ during exercise testing in individuals with stroke. Our findings could potentially contribute to the development of appropriate therapies to improve cardiorespiratory fitness in individuals with stroke.
